# On Pulmonary Diseases

**Published:** 1816-03

**Authors:** Thomas Sutton


*For the London Medical and Physical Journal.*

### *On Pulmonary Diseases;* by Thomas Sutton, M.D.

As I perceive, in Dr. Buxton's last Letter, nothing that needs so particular a reply as to give to this communication the form of controversy, and as every end I could have in view has been already answered in so far as relates to him, I shall, at present, wave, as much as possible, any allusion to that gentleman or his institution. I must, however, assure the doctor, that my object has not been a victory over him, or personally over any one, but a wish to rectify some errors that appear to me to have crept into the practice respecting Consumption; and to excite to further, and, I hope, more accurate, observation on the subject, both in so far as the treatment of the disease is concerned, and also in respect to some facts and analogical conclusions which I judge to be in need of re-consideration. I shall now direct this and any future communication I may make on the present subject to these objects.

Having stated that the air of closed apartments, and high equable artificial temperature, are not favourable to diseases in the form of pulmonary consumption, I shall begin by giving some cases somewhat bearing on that subject.

Case I.—This is the case of a young lady, aged 20, who was under the care of Mr. Emerson, of Sandwich, and communicated to me by letters, the first of which I have abridged somewhat, retaining, however, every material feature of the case. The patient was attacked suddenly on the 18th of last January, with pneumonia of the most acute kind, but chiefly confined to the left side. Ten ounces of blood were immediately taken away, and on the following day eight ounces: the strictest antiphlogistic regimen was enjoined. On the active state of inflammation being subdued, no decided symptoms of amendment took place; but, in the course of a month, hectic symptoms seemed approaching, accompanied by an expectoration which had every character of purulence, in quantity sometimes exceeding one one pint and a half in twenty-four hours. The pulse at this time was 100 and upwards. A portion of the expectoration was now dissolved in vitriolic acid, according to Mr. C. Darwin's experiments, which, on the addition of water, threw down only a small precipitate. The symptoms were, however, now sufficiently alarming to induce Mr. E. to request a consultation with a physician in the neighbourhood. The plan agreed on was to use a more tonic and astringent diet; to continue in the use of digitalis; and to employ gentle sedatives and chalybeates to strengthen and to allay irritation. On this plan, the patient, after the expiration of a month, became improving. The pulse was reduced to 60 and 70, and natural in its pulsations; the perspiration diminishing; cough and expectoration abating, and both in a short time quite disappeared; increasing appetite and strength, with tranquil sleep, indicated returning health. Without any apparent cause, in the latter end of March, every symptom of the disease, only in a lesser degree, returned, and which was succeeded by the same progress to amendment, viz. the disappearance of cough, expectoration, and perspiration, &c. This amendment continued to the latter end of April, when a relapse ensued, and is now followed by the same apparent recovery.

The letter stating the above particulars is dated the 12th of May, in which Mr. E. also says, "my patient, as you may naturally suppose, after such repeated attacks, is much emaciated." In another part of the letter he states her strength to be equal to being able to sit up "an hour or two."—"During the progress of the disease, the menses have only appeared twice, at irregular periods." An uniform and medium temperature has been attempted; and, in a room uninfluenced by artificial heat, during the last two months, the thermometer has averaged 63°." Mr. Emerson also asks, "Can a more probable cause be suggested for these fluctuations than the formation of vomicæ or circumscribed abscesses?"

In answer to this letter, I recommended that the patient should be allowed to breathe as much of the external air as possible; and that, instead of keeping the room at 63°, it should, at that time of year, be kept in as low a temperature as could be obtained. For both which purposes, I recommended the windows to be kept open as much as could be by night and by day; that the patient should be every day brought into the garden, when the weather would admit, and placed in the shade, and remain there as long as her strength would allow. On the subject of diet, I recommended mild nutritive food to be given, and some tender butcher's butcher's meat; to take the principal meal early, and in that case to drink a glass of wine diluted with water. On the subject of medicine, I recommended the bowels to be kept moderately open, the cough to be restrained by the use of opium; and, in case the pain in the side should return, to try the effects of a cold lotion to the part; and avoid bleeding, if possible. In about a month I heard from the father, a clergyman, that his daughter was much improved in health; and, some time afterwards, Mr. Emerson sent me a letter on the subject, all of which, in so far as relates to this case, I transcribe—

"DEAR SIR,

"I feel much satisfaction in being able to corroborate the statement which I learn you have received, through my patient's father, respecting her health; whose amendment, since I had the pleasure of writing to you on the subject, under a partial obedience to the plan you suggested, has been progressive, and which has, indeed, proceeded to that degree, that, with due care and restraint, I have no hesitation in supposing will prove permanent. No inflammatory symptoms recurring, the cold application has not been applied, but the free admission of cool air into the room, moderate exercise in the garden, with a full opiate at bed-time, and slight tonic medicines and diet, has been the plan pursued. Medicine has, however, been gradually relinquished, and her health so far restored, as now to be almost totally abandoned. The appetite is sufficiently good; her strength and size increasing, and the former so much improved as to enable her to go through the day without rest, and to ride two or three miles on a mule without fatigue. The chest seems quite free from the least irritation; the pulse has become natural; and her sleep is tranquil, and equal to perfect health. To this apparent safety has my patient arrived from great *danger* and even *despair*. For your judicious suggestions I feel much obliged.

(Signed) R. Emerson, jun."

*Sandwich; June* 28, 1815.

I received a letter from the father of this patient, dated Nov. 29th, stating that she was gone to Huntingdonshire, and says, his daughter's report of herself is, in every respect, satisfactory. He adds, "I never witnessed such a recovery, and am grateful to Providence and to you."

Case II.—A man, about the age of forty, became a patient of the Kent Dispensary about June last: he had previously been attended by Mr. Bromley, one of the surgeons to this Dispensary, as a private patient. When I saw him, he shewed considerable marks of progress in emaciation: he had a constant irritating cough, with expectoration; a pulse of 120 and upwards; night perspirations; much heat on the skin, skin; a constant teasing pain on one side; and a very frequent disposition to vomit. Mr. Bromley also stated, that, in consequence of his sensations of chilliness, he had often seen the patient very near the fire, and coveting external warmth. In respect to medicines, I found no need to vary the plan adopted, being moderately aperient, soothing, and demulient. Cold lotion had also been used to the chest; but it was now strongly enjoined that the patient should seek the greatest degree of cold he could, and to respire the external air as much as possible. For this purpose, I recommended him to be as much out of doors, in the shade, as he well could be; to avoid the heat of the sun; and to sleep with his chamber-windows unclosed, and his bed quite open, while he applied the lotion to the chest, and pursued the plan of medicine before stated. I felt no hesitation in allowing him a small portion of animal food when his stomach would bear it, and a small quantity of porter, provided he adhered to the plan of temperature and air I had enjoined. In ten days I again saw him, when the countenance was considerably amended. Instead of the heated flush of the face, it was rather pallid, indicating a severe or length of illness: his pulse was 84; his cough, expectoration, and pain in the side, had diminished much; and he had little or no perspiration. In a space of time from his adopting this plan, not exceeding, I believe, five weeks, he desired to be discharged from the Dispensary, considering himself as quite well. At this time Mr. Bromley said he had not entertained the least notion of the man's recovery when he became a patient of the Dispensary. This I state as an abundant proof that the case wore a very threatening and unpromising aspect. I have every reason to believe the patient followed the advice given him with the greatest strictness; and ought to add, that the man was reported to me to be addicted to inebriety.

Case III.—I visited, about the middle of August last, the son of an opulent family residing near Farningham. He was reported to me to have been attacked, somewhat more than two months ago, with a fever, accompanied by pulmonary affection, while at a distance from his friends, for which one of the remedies was blood-letting; and, as soon as the inflammatory symptoms had somewhat abated, he was removed to a short distance for change of air, as he continued weak, and made no progress in recovery. Lastly, he was sent home, the uneasiness in his chest continuing, accompanied by cough, with an injunction to be very guarded respecting the external air and cold. In the week or ten days before I saw him, the cough and pain in the side had so much increased as to render venesection twice necessary, with the application of a blister to the part affected. He also began to have night perspirations, and was grown much thinner. I found the patient with a quick pulse, a flushed countenance, complaining of pain in his chest and uneasiness in respiration, and with a considerable dread of the cold and external air. After some conversation, however, with him, I persuaded him to dismiss all fears of that sort, provided he used a prudent and persevering conduct in respect to them. I recommended him to sleep with his window open, to be as much as possible in the open air in the shade, and, when the blister was well, or even before it was healed, to apply cold lotion to his chest. As the bowels were inclined to be bound, the principal remedy I recommended was a purgative in the Infus. Rosæ. In a fortnight from this, my patient paid me a visit at Greenwich early in the morning, in an open chaise, and expressed his grateful thanks for the happy change wrought in his health through my advice. He afterwards went to Brighton for a short time, and returned in good health. Mr. Edwards, surgeon, of Farningham, who attended the patient, informed me that every thing I recommended was strictly complied with. The brother of this young gentleman, who is a few years older, has strong marks of incipient consumption. The age of the patient whose case I have given was 19.

Case IV.—The son, aged eight years, of a gentleman in the neighbourhood of Bexley, was attacked, about the beginning of last May, with measles, with considerable pulmonary affection, and which was so soon followed by the hooping cough, as, in fact, to warrant the conclusion that he had both the diseases at the same time, as the latter strongly evidenced its existence before the measles were over. In the latter end of the month I was sent for to visit him, having also once seen him in the measles. At this period, from a fine stout child, he was reduced to the merest skeleton, and in the greatest state of debility, with a pulse of 140 and upwards. He had lost the peculiar noise of the hooping cough, but expectorated a puriform matter to the quantity of an ounce at a time, and had much heat, but with an increase of fever at night, followed very frequently by considerable perspirations. The child had hitherto been nursed in a close and warm apartment; his body was enclosed in flannel; and great fears were entertained by the family of the disease being increased by cool air, or the access of the external atmosphere. The matter expectorated appeared to give strong evidence of an abscess having formed in the lungs: the reduced and weak state of the child were very unpromising, together with the circumstance of the family being prone to consumption; and one of the children of this family had also died, at about the age of the present patient, of a pulmonary affection following the hooping cough and measles, which was, in its prominent circumstances, considered to be so like to the present, as to cause the family to consider this case also as incapable of recovery. Under these unfavourable circumstances, I, however, recommended a total change in regard to temperature, clothing, and air. I requested the flannel to be quickly but gradually removed; that the child be without fire, in a room of considerable size, which the house could well supply, and to be removed from it if the sun at any time shone powerfully on it; to be with the windows open both by night and by day; to sleep without the curtains being drawn round the bed; and to be by day as much in the open air as possible, and in the shade. All this advice was strictly attended to, and the child was taken out in the carriage for several hours in the day time, with the windows down. For the ten first days there was no very obvious amendment, although nothing, in any respect, worse; but, at the beginning of the third week, the child was evidently better, and was considered to be out of danger by the family in the fourth week, and he afterwards rapidly lost all urgent symptoms. He is now a fine stout boy. In respect to medicine, the bowels were attended to, and soothing demulient medicines were given, and varied according to circumstances. This young gentleman was a patient of Mr. Francis, surgeon, of Bexley.

These cases I more particularly relate, because they were attended by respectable surgeons, who, on their part, did every thing to encourage the parties concerned to adopt and continue the plan which was recommended to them. I may here also take the opportunity of saying, that I have the happiness of being generally connected with practitioners of such liberal minds and justness of information, as to be ready to co-operate in every effort recommended for the good of their patients, when candidly explained, and the motives and views properly exhibited. Thus the experience of facts, and the history of events, become doubly important, as I can generally depend upon the account of those surgeons with whom I am connected, with as much confidence as if each individual circumstance passed distinctly under my own observation.

Of the cases, however, which I have given, I do not wish any one of them to be considered as a case of true pulmonary consumption, if such as originate from tubercles alone are agreed to be called so, to which, for method's sake, I should

 entertain entertain no objection. Of these cases, I believe, none recover. I would, then, call all cases with symptoms of pulmonary consumption, not so originating, spurious pulmonary consumption, the latter of which I believe to be the greatest number which take this name, and the greater part of which, I think, are capable of cure. These last diseases are often more or less connected with tubercles; and, in the progress of a case of spurious consumption, I believe the lungs prone to take on a tubercular state, as I have seen fatal cases of this kind evidently commencing through accidental disease, when investigated, to exhibit the formation of tubercles, though some to no great extent. It will also happen often that a spurious consumption in its commencement may be afterwards connected with a tubercular one, or become a mixed case in such proportion as the subject may be prone to tubercles in the lungs. I also conclude, that there are many fatal cases of spurious consumption in this country, that, by a different method than that usually adopted and countenanced, might be saved. In addition to what I have said in confirmation of this opinion, the facts immediately following may deserve consideration.

To prove the greater fatality of consumption in the colder months of the year in this country, a statement was made in your Journal, from the Bills of Mortality in London. No. 5–12, including the eight weeks from the 11th of January, 1814, to the 8th of March, give 947 deaths by consumption. No. 29–36 of the same year, from the 28th of June to the 23d of August, give 585 deaths by the same disease, making an excess of deaths in winter to the summer months of more than one-third. Bayle, physician to the Charité at Paris, who has devoted much time and attention to consumption, has given a table of the time of year of the deaths of 244 patients, during three years, in two of the medical wards of that institution, which he sums up as follows:—64 died in autumn—58 in winter—54 in spring—68 in summer. He no-where in his books contradicts or doubts this result, in regard to his private practice, nor hints at the greater frequency of deaths in this disease in winter than in summer, which could not have escaped the attention of a man so devoted to this enquiry, had the ratio of deaths in his practice in any part of the year exceeded any other in the proportion it appears to do in this country. This great inequality of result, then, in regard to the greater fatality of the disease in winter among us than happens in France, must be owing to some cause very materially affecting the disease. This I am disposed to attribute to the greater attention to warmth in the treatment of the disease in this country, country, in so far as it regards apartments, which acts to the exclusion of the external atmosphere, the fear of breathing the external air, and the factitious warmth employed, together with the numerous means of rendering apartments close in this country which are not thought of in France. Indeed the French are habituated to endure a greater degree of cold, on account of the materials for fire being less abundant and more expensive than with us, and such as they have, and the methods of using them, not tending to so permanent a warmth as with us.

Bayle also gives a table of the comparative number of deaths by consumption in the two wards of the Charité at Paris, during three years, which shews a greater fatality by this disease at Paris than in this country, or the great disadvantages, in respect to their recovery, which consumptive patients labour under in an institution in which they are placed with a considerable number of other patients: to the latter cause I must attribute the unexampled relative mortality alluded to. The total number of deaths by this account were 696, of whom 244 died of phthisis, which is in the ratio of deaths of more than one in three by the latter disease.

I shall conclude this communication by remarking, that our knowledge in England of the ravages of consumption in other countries is very incomplete, as is evidenced by the above table; and, although we should be inclined to admit that many of these cases might have been saved by being placed under other circumstances than in a public hospital, together with the consideration that the death of surgical cases are not included, yet then the mortality in Paris in consumption will exceed much that which has been generally believed in this country to have happened there, if these latter considerations should be supposed to reduce the mortality by one half.

I have also been obliged by having the contents of the bills of mortality* for St. Petersburgh, &c. translated for me, from the Russian Almanack for 1815, by favour of General Sablanhaff, in which the mortality by consumption and pulmonary disease appears to exceed one in five. As this abstract is, I am persuaded, very carefully drawn up, and contains more interesting particulars, I send it to you, with a recommendation to print it, with a hope, at the same time, that you will endeavour, as much as possible, to obtain si-

* We gave place to the above-mentioned Bills in the Intelligence of our last Number.—Edit.

milar milar documents of mortality, &c. from other countries, which, as containing matter of general information, must always be agreeable to your readers, and may also tend to assist many useful inquiries respecting disease, &c.

*Croom's-hill, Greenwich;*

*Dec.* 31, 1815.

